# *Portulaca oleracea* L.: A Review of Phytochemistry and Pharmacological Effects

**DOI:** 10.1155/2015/925631

**Published:** 2015-01-26

**Authors:** Yan-Xi Zhou, Hai-Liang Xin, Khalid Rahman, Su-Juan Wang, Cheng Peng, Hong Zhang

**Affiliations:** ^1^Key Laboratory of Standardization of Chinese Herbal Medicines of Ministry of Education, Pharmacy College, Chengdu University of Traditional Chinese Medicine, Chengdu 610075, China; ^2^Central Laboratory, Shanghai Seventh People's Hospital, Shanghai 200137, China; ^3^Department of Traditional Chinese Medicine, Changhai Hospital, Second Military Medical University, Shanghai 200433, China; ^4^Department of Pharmacognosy, School of Pharmacy, Second Military Medical University, Shanghai 200433, China; ^5^School of Pharmacy and Biomolecular Sciences, Faculty of Science, Liverpool John Moores University, Liverpool L3 3AF, UK; ^6^Department of Pharmaceutical Botany, School of Pharmacy, Second Military Medical University, Shanghai 200433, China

## Abstract

*Portulaca oleracea* L., belonging to the Portulacaceae family, is commonly known as purslane in English and Ma-Chi-Xian in Chinese. It is a warm-climate, herbaceous succulent annual plant with a cosmopolitan distribution. It is eaten extensively as a potherb and added in soups and salads around the Mediterranean and tropical Asian countries and has been used as a folk medicine in many countries. Diverse compounds have been isolated from *Portulaca oleracea*, such as flavonoids, alkaloids, polysaccharides, fatty acids, terpenoids, sterols, proteins vitamins and minerals. *Portulaca oleracea* possesses a wide spectrum of pharmacological properties such as neuroprotective, antimicrobial, antidiabetic, antioxidant, anti-inflammatory, antiulcerogenic, and anticancer activities. However, few molecular mechanisms of action are known. This review provides a summary of phytochemistry and pharmacological effects of this plant.

## 1. Introduction

*Portulaca oleracea* L. is a warm-climate, herbaceous succulent annual plant with a cosmopolitan distribution belonging to the Portulacaceae family. It is commonly known as purslane (USA and Australia), rigla (Egypt), pigweed (England), pourpier (France), and Ma-Chi-Xian (China) [1]. It is distributed widely in the tropical and subtropical areas of the world including many parts of the United States and is eaten extensively as a potherb and is added to soups and salads around the Mediterranean and tropical Asian countries [2]. Americans and aborigines of Australia grind the seeds of this plant into flour for use in mush and bread [3].* Portulaca oleracea* also provides a source of nutritional benefits owing to its rich omega-3 fatty acids and antioxidant properties [4].

*Portulaca oleracea *has been used as a folk medicine in many countries, acting as a febrifuge, antiseptic, vermifuge, and so forth [5]. It exhibits a wide range of pharmacological effects, including antibacterial [6], antiulcerogenic [7], anti-inflammatory [8], antioxidant [9], and wound-healing [10] properties. It is listed by the World Health Organization as one of the most used medicinal plants, and it has been given the term “Global Panacea” [11]. The Chinese folklore described it as “vegetable for long life” and it has been used for thousands of years in traditional Chinese Medicine [12, 13]. It is cold in nature and sour in taste and is used to cool the blood, stanch bleeding, clear heat, and resolve toxins. The dried aerial part of this plant is indicated for the treatment of fever, dysentery, diarrhoea, carbuncle, eczema and hematochezia, with a recommended dose of 9–15 g [14–16].

*Portulaca oleracea* has a high potential to be used as human and animal food and to be utilized as a pharmacological agent in medicine. In this paper, phytochemistry and pharmacological activities of this plant are reviewed and its potential for further investigation, exploitation, and utilization are discussed.

## 2. Phytochemistry

**Figure 1:**
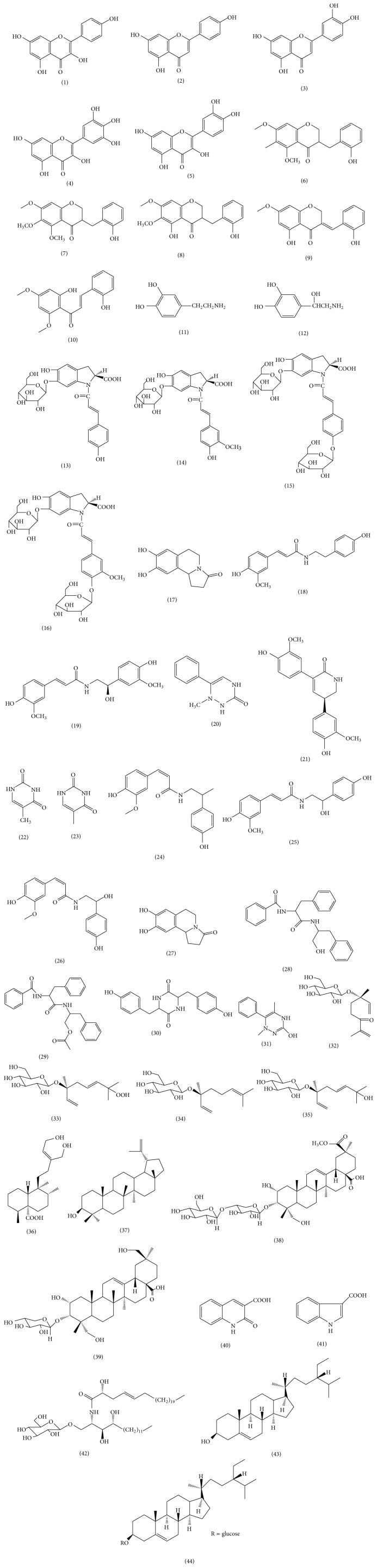
Chemical structures of main compounds present in* Portulaca oleracea*.

Many constituents of* Portulaca oleracea* have been isolated, including flavonoids, alkaloids, fatty acids, terpenoids, polysaccharides, vitamins, sterols, proteins, and minerals; these are listed in Table 1 and the chemical structures of the main compounds are presented in Figure 1.

One of the most effective constituents present in Chinese Herbal Medicines are flavonoids which are biologically active and possess a wide range of pharmacological properties such as antibacterial, antivirus, anti-inflammation, and antioxidation properties. In the* Portulaca oleracea* plant, the flavonoids levels vary according to the part of the plant; the highest levels are present in the root followed by stem and the leaf; and seven different flavonoids are present in this plant, including kaempferol, myricetin, luteolin, apigenin, quercetin, genistein, and genistin [17]. However, only kaempferol and apigenin have been found in ethanolic extracts of leaves and stems, with the levels in the former being higher [11]. Portulacanones B–D, three homoisoflavonoids compounds, display selectively cytotoxic activities against three human cancer cell lines (SF-268, NCI-H460, and SGC-7901) [18]. Flavonoids are also widely present in foods such as fruits and vegetables [19].

In addition to flavonoids, another important chemical found in this plant is alkaloids including dopa, dopamine, and noradrenalin. The content of dopamine and noradrenalin is higher in leaves compared to stem and seeds. The amount of dopamine and noradrenalin obtained from leaves varies according to the solvents used in the extraction process, suggesting that the levels of these compounds are dependent on the solvents used during the extraction process [20]. Oleraceins A, B, C, D, and E are cyclodopa alkaloids isolated from this plant [21] and several analytes such as (3R)-3,5-bis(3-methoxy-4-hydroxyphenyl)-2,3-dihydro-2(1H)-pyridinone and 1,5-dimethyl-6-phenyl-1,2-dihydro-1,2,4-triazin-3(2H)-one display cytotoxic activities against human cancer cells [22].

*Portulaca oleracea* is also an excellent source of omega-3 fatty acids, which is usually present in oil and fat of fishes but not normally found in plants. Omega-3 fatty acids play an important role in the enhancement of immune function [23] and prevention and treatment of hypertension, coronary artery disease, cancer, and other inflammatory and autoimmune disorders [24]. It includes *α*-linolenic acid and linoleic acid, which are essential for normal growth, health promotion, and disease prevention in humans. Polysaccharides found in* Portulaca oleracea* are potential therapeutic agents for the treatment of diabetes mellitus owing to their modulation of blood lipids, metabolism, and decrease of blood glucose.* Portulaca oleracea* contains monoterpenes such as portulosides A and B, diterpenes such as portulene, and *β*-amyrin type triterpenoids [1, 25]; in addition, vitamins have also been isolated from the leaves of this plant. It contains the highest content of vitamin A which is a natural antioxidant playing an important role in vision, maintaining healthy mucus membranes and protecting against lung and oral cavity cancers among green leafy vegetables. This plant also contains ascorbic acid, *α*-tocopherol, and B-complex vitamins, for example, niacin, pyridoxine, and riboflavin [26]. Furthermore it is rich in minerals like phosphorus, manganese, icon, calcium selenium [3], and the amino acids isoleucine, proline, leucine, lysine, phenylalanine, methionine, cystine, valine, threonine, and tyrosine [2]. Many other constituents have also been isolated from this plant, such as *β*-carotene, glutathione, melatonin, portulacerebroside A, catechol, and bergapten.

## 3. Pharmacology

Over the past decades, numerous researchers have investigated the pharmacological activities of* Portulaca oleracea*. This review provides a comprehensive summary of the main pharmacological properties which are presented below.

### 3.1. Neuroprotective Activity

Administration of* Portulaca oleracea* can scavenge free radicals and antagonize rotenone-induced neurons apoptosis, dopamine depletion, and complex-I inhibition in striatum of rats, suggesting that* Portulaca oleracea* may be a potential neuroprotective candidate against Parkinson's disease [23]. The extract of* Portulaca oleracea* (EP) protects nerve tissue/cells from hypoxic damage probably by elevation of glycolysis, EPO, and hypoxia inducible factor-1 expression levels [27]. The ethanol extract decreases the activity of caspase-3 in neuron whilst reducing serum levels of neuron specific enolase in hypoxia mice and the pathological damages caused by hypoxia. In these studies, an increase in the neuron viability and an induction in the mRNA and protein expression of endogenous erythropoietin have also been reported. Thus, the stabilization of hypoxia inducible factor-1 *α* expression is associated with the neuroprotective effects of EP against hypoxia injury by eliciting endogenous erythropoietin expression [28]. *β*-Cyanin evidently inhibits D-galactose-induced neurotoxicity in mice, which at the doses of 50 and 100 mg/kg upregulates the activities of superoxide dismutases, catalase, glutathione reductase, and glutathione peroxidase, whilst reducing the level of the lipid peroxidation product malondialdehyde in the brain of D-galactose-treated mice. When compared to vitamin C, *β*-cyanin play a more pronounced effect on alleviating cognition deficits in mice [29]. The total alkaloidal extracts from 31 traditional Chinese Herbal Medicines were tested for their acetylcholinesterase (AChE) inhibitory activities by Ellman's method and modified TLC bioautographic assay. As a result, the alkaloidal extract of* Portulaca oleracea* significantly inhibited AChE activity at a final concentration of 100 *μ*g/mL with the IC50 value of 29.4 *μ*g/mL. The use of AChE inhibitors has been a promising treatment strategy for Alzheimer's disease (AD); therefore,* Portulaca oleracea* may be an effective agent for the prophylaxis and treatment of AD [30].

### 3.2. Antidiabetic Activity

*Portulaca oleracea* attenuates body weight, serum free fatty acids, and hyperinsulinemia. It also increases insulin sensitivity and ameliorates impaired glucose tolerance and lipid metabolism in rats with type 2 diabetes mellitus induced by injection of streptozotocin (25 mg/kg) and feeding of high calorie forage, suggesting that* Portulaca oleracea* alleviates insulin resistance [31]. Administration of the seeds powder (5 g × 2/day) increases high density lipoprotein cholesterol (HDLC) and albumin, while lowering the levels of serum total cholesterol, triglycerides, low density lipoprotein cholesterol (LDLC), liver gamma glutamyl transaminase (GGT), alanine transaminase (ALT), aspartate transaminase (AST), total and direct bilirubin, fasting and postprandial blood glucose, insulin, body weight, and body mass index in type 2 diabetic subjects. There were no differences in these results compared to the data obtained with metformin treatment (1500 mg/day) except for LDLC, HDLC, and alkaline phosphatase (ALP) levels, suggesting that* Portulaca oleracea* seeds are valuable and effective as an adjunctive and alternative therapy for the treatment of type 2 diabetes mellitus [32].

The aqueous extract of* Portulaca oleracea* also prevents diabetic vascular inflammation, hyperglycemia, and diabetic endothelial dysfunction in type 2 diabetic db/db mice, suggesting its protective role against diabetes and related vascular complications [33]. The crude polysaccharide extract of this plant also lowers blood glucose and modulates the metabolism of blood lipids and glucose in alloxan-induced diabetic mice [34], whilst decreasing the levels of total cholesterol, triglycerides, and fasting blood glucose in type 2 diabetic mice [32].

### 3.3. Antioxidant Activity

The antioxidant property of* Portulaca oleracea* is attributed to its constituents, such as gallotannins, omega-3 fatty acids, ascorbic acid, *α*-tocopherols, kaempferol, quercetin, and apigenin [8, 16, 17]. The single cell gel electrophoresis assay (comet assay), which is an simple, rapid, and inexpensive method for measuring DNA strand breaks, confirmed that the aqueous extract significantly alleviated hydrogen peroxide-induced oxidative DNA lesions in human lymphocytes, whereas the ethanolic extract had no effects, which may be associated with the antioxidant constituents contained in the aqueous extract [35]. The aqueous extract decreases high fat diet-elicited oxidative damage by modulating blood and liver antioxidant enzyme activities, elevating leptin/*β*-actin and liver PPAR a/*β*-actin and inhibiting the protein expression of p-PERK and the FAS mRNA expression of liver and spleen in mice [9]. In another study, the aqueous extract at a concentration range of 100, 150, 200, and 400 *μ*g/mL and the ethanolic extract at a range of 1200 and 1800 *μ*g/mL, respectively, exerted cytoprotective effects on 2,2′-azobis hydrochloride-induced hemolytic damages of RBCs in a concentration-dependent manner [36].

### 3.4. Anticancer Activity

Polysaccharides from* Portulaca oleracea* display several biological activities, such as anticancer, antioxidation, anti-inflammation, and immunity enhancing properties [37–40]. Polysaccharides evidently scavenge the accumulation of free radicals and modulate immunity functions of rats with ovarian cancer [41]. Sulfated derivatives of POP, a water-soluble polysaccharide isolated from* Portulaca oleracea*, have a suppressive effect on the growth of HeLa and HepG2 cells* in vitro*, suggesting that the sulfation of POP increases the cytotoxicity in tumor cells [42]. In addition to polysaccharides, other bioactive compounds such as cerebrosides, homoisoflavonoids, and alkaloids also show* in vitro* cytotoxic activities against human cancer cell lines. Portulacerebroside A stimulates human liver cancer HCCLM3 cell apoptosis via the activation of the p38 MAPK- and JNK-triggered mitochondrial death pathway [43] and 2,2′-dihydroxy-4′,6′-dimethoxychalcone is more active against cell line SGC-7901 with an IC_50_ value of 1.6 ug/mL than mitomycin C which has an IC_50_ value of 13.0 ug/mL. Portulacanones B is active against SGC-7901 cell lines with an IC_50_ value of 16.2 ug/mL, which is very close to the value obtained with mitomycin C. 2,2′-Dihydroxy-4′,6′-dimethoxychalcone is moderately active against K-562 cells with an IC_50_ value of 24.6 ug/mL and portulacanones B–D show selective cytotoxic activity against SF-268 and/or NCI-H460 cells with IC_50_ values of 14.3–20.1 ug/mL [18]. N-*trans*-Feruloyltyramine, (7′R)-N-feruloylnormetanephrine, 1,5-dimethyl-6-phenyl-1,2-dihydro-1,2,4-triazin-3(2H)-one, and (3R)-3,5-bis(3-methoxy-4-hydroxyphenyl)-2,3-dihydro-2(1H)-pyridinone have weak bioactivities against K562 with IC_50_ values of 222.77, 66.94, 90.09, and 41.52 umol/L, respectively, and moderate bioactivities against A549 with IC_50_ values of 28.80, 21.76, 24.54, and 37.20 umol/L, respectively [22]. These studies demonstrate that* Portulaca oleracea* has a potential application in the treatment of cancer.

### 3.5. Antimicrobial

*Portulaca oleracea* possesses antibacterial, antifungal, and antiviral activities as revealed by its antifungal effect against dermatophytes of the genera* Trichophyton* [44]. A pectic polysaccharide isolated from the aerial part of this plant displays antiherpes property against simplex virus type 2 which is due to the inhibition of virus penetration and not virus adsorption [45]. A 70% methyl alcohol extract of* Portulaca oleracea* shows antibacterial activity against the Gram-negative stains:* Escherichia coli*,* Pseudomonas aeruginosa,* and* Neisseria gonorrhea* with inhibition zones of 14, 15, and 15 mm, respectively, and the Gram-positive strains:* Staphylococcus aureus*,* Bacillus subtilis,* and* Streptococcus faecalis* with inhibition zones of 13, 14, and 15 mm, respectively, as well as antifungal activity against* Candida albicans* with inhibition zone of 12 mm [1].

### 3.6. Anti-Inflammatory Activity

Pretreatment with the aqueous extract of* Portulaca oleracea* inhibits tumor necrosis factor- (TNF-) *α*-induced production of intracellular reactive oxygen species (ROS) and overexpression of intercellular adhesion molecule- (ICAM-) 1, vascular cell adhesion molecule (VCAM)-1, and E-selectin in human umbilical vein endothelial cells (HUVECs) in a dose-dependent manner. This extract also suppresses the translocation of nuclear factor *κ*B (NF-*κ*B) p65 to the nucleus, TNF-*α*-induced NF-*κ*B binding, and the degradation of inhibitor molecule (I*κ*B)*α*. Furthermore, an inhibition in the adhesion of HL-60 cells to TNF-*α*-induced HUVECs and TNF-*α*-induced mRNA expression of interleukin- (IL-) 8 and monocyte chemoattractant protein- (MCP-) 1 is also observed. The aqueous extract of* Portulaca oleracea* may also play an important role in the suppression of the vascular inflammatory process related to the development of atherosclerosis [46].

### 3.7. Antiulcerogenic Activity

Aqueous and ethanolic extracts of* Portulaca oleracea* at 0.8 g/kg and 1.4 g/kg, respectively, can reduce the severity of HCl-induced gastric ulcers in a dose-dependent manner; this is comparable to the effect observed with sucralfate 0.1 g/kg. In addition, the aqueous extract (0.56 and 0.8 g/kg) and the ethanolic extract (0.8 and 1.4 g/kg) display suppression of lesions induced by absolute ethanol. The oral and intraperitoneal doses of both extracts dose-dependently increase the pH of gastric juice in mice with pylorus ligation. Thus,* Portulaca oleracea* holds great promise as an effective therapeutic agent for gastrointestinal diseases due to its gastroprotective activity [7].

### 3.8. Hepatoprotective Activity

Intraperitoneal administration of CCl_4_ elicits liver injury in rats, which notably upregulates the levels of total bilirubin and serum hepatic marker enzymes, including glutamate pyruvate transaminase (GPT) and glutamate oxaloacetate transaminase (GOT). A 70% alcohol extract of* Portulaca oleracea* significantly reverses the increase in hepatic marker enzymes and total bilirubin levels, confirming the hepatoprotective activity of this plant [1].

### 3.9. Other Activities

The ethanol extract from* Portulaca oleracea* at a concentration range of 100, 200, and 400 mg/kg, respectively, displays a dose-dependent effect in prolonging the survival time of mice in hypoxic models, including closed normobaric hypoxia and potassium cyanide or sodium nitrite toxicosis. This extract also enhances the activities of phosphofructokinase, pyruvate kinase, and lactate dehydrogenase in glycolysis and the level of adenosine triphosphate of mouse cortices in hypoxia models [12]. The preliminary wound healing activity of* Portulaca oleracea* has been appraised in* Mus musculus *JVI-1 and it has been shown that a fresh crude extract significantly accelerates the wound healing course by the stimulation of wound contraction and downregulation of the surface area of the excision wound [10].* Portulaca oleracea* also has the ability to accumulate Se even at the shortest time span of 42 days, and hence it can perform the dual functions of preventing the occurrence of Se deficiency linked diseases such as Keshan and Kashin-Beck diseases [47].

## 4. Conclusion

*Portulaca oleracea* is of considerable importance to the food industry and also possesses a wide spectrum of pharmacological properties such as neuroprotective, antimicrobial, antidiabetic, antioxidant, anti-inflammatory, antiulcerogenic, and anticancer activities, which are associated with its diverse chemical constituents, including flavonoids, alkaloids, polysaccharides, fatty acids, terpenoids, sterols, proteins, vitamins, and minerals.

Although bioactivities of extracts or compounds isolated from* Portulaca oleracea* are substantiated by using* in vitro* and* in vivo* studies including animal models and cell culture studies, the mechanisms of action have not been addressed. Hence, more mechanistic studies are required before* Portulaca oleracea* can be considered for further clinical use. This review concludes that* Portulaca oleracea* is an edible and a medicinal plant which is important to the food industry and may also have a significant role to play in health care provided that adequate studies are conducted.

## Figures and Tables

**Table 1:** Compounds isolated from purslane.

Classification	Chemical component	Part of plant	Reference
Flavonoids	Kaempferol (1)	Leaf and stem	[11]
	Apigenin (2)	Leaf and stem	[11]
	Luteolin (3)	Whole plant	[11]
	Myricetin (4)	Whole plant	[11]
	Quercetin (5)	Whole plant	[11]
	Portulacanones A (6)	Aerial part	[18]
	Portulacanones B (7)	Aerial part	[18]
	Portulacanones C (8)	Aerial part	[18]
	Portulacanones D (9)	Aerial part	[18]
	2,2′-Dihydroxy-4′,6′-dimethoxychalcone (10)	Aerial parts	[18]
	Genistein	Whole plant	[17]
	Genistin	Whole plant	[17]

Alkaloids	Dopamine (11)	Stem, leaf and seed	[20]
	Noradrenalin (12)	Stem, leaf and seed	[48]
	Dopa		[21]
	Oleraceins A (13)	Whole plant	[21]
	Oleraceins B (14)	Whole plant	[21]
	Oleraceins C (15)	Whole plant	[21]
	Oleraceins D (16)	Whole plant	[21]
	Oleraceins E (17)	Whole plant	[21]
	Oleracins I	Stem	[21]
	Oleracins II	Stem	[21]
	Adenosine	Whole plant	[21]
	*N*-*trans*-Feruloyltyramine (18)	Aerial part	[22]
	(7′R)-*N*-Feruloylnormetanephrine (19)	Aerial part	[22]
	1,5-Dimethyl-6-phenyl-1,2-dihydro-1,2,4-triazin-3(2H)-one (20)	Aerial part	[22]
	(3R)-3,5-Bis(3-methoxy-4-hydroxyphenyl)-2,3-dihydro-2(1H)-pyridinone (21)	Aerial part	[22]
	Thymine (22)	Aerial parts	[18]
	Uracil (23)	Aerial parts	[18]
	*N*-*cis*-Feruloyltyramine (24)	Aerial parts	[18]
	*N*-*trans*-Feruloyloctopamine (25)	Aerial parts	[18]
	*N*-*cis*-Feruloyloctopamine (26)	Aerial parts	[18]
	Trollisine (27)	Aerial part	[49]
	Aurantiamide (28)	Aerial part	[49]
	Aurantiamide acetate (29)	Aerial part	[49]
	Cyclo(L-tyrosinyl-L-tyrosinyl) (30)	Aerial part	[49]
	1,5-Dimethyl-6-phenyl-1,6,3,4-tetrahydro-1,2,4-2(1H)-triazin (31)	Aerial part	[49]
	Scopoletin		[50]

Terpenoids	Portuloside A (32)	Aerial part	[51]
	Portuloside B (33)	Aerial part	[52]
	(3*S*)-3-O-(*β*-D-Glucopyranosyl)-3,7-dimethylocta-1,6-dien-3-ol (34)	Aerial part	[52]
	(3*S*)-3-O-(*β*-D-Glucopyranosyl)-3,7-dimethylocta-1,5-dien-3,7-diol (35)	Aerial part	[52]
	Portulene (36)	Aerial part	[1]
	Lupeol (37)	Aerial part	[1]
	(2*a*,3*a*)-3-{[4-O-(*β*-D-Glucopyranosyl)-*β*-D-xylopyranosyl]oxy}-2,23-dihydroxy-30-methoxy-30-oxoolean-12-en-28-oic acid (38)	Aerial part	[25]
	(2*a*,3*a*)-2,23,30-Trihydroxy-3-[(*β*-D-xylopyranosyl)oxy]olean-12-en-28-oic acid (39)	Aerial part	[25]
	Friedelane	Aerial part	[25]

Organic acids	3-Quinolinecarboxylic acid (40)	Aerial parts	[18]
	Indole-3-carboxylic acid (41)	Aerial parts	[18]
	a-Linolenic acid	Leaf	[24]
	Linoleic acid	Leaf	[26]
	Palmitic acid	Leaf	[4]
	Stearic acid	Leaf	[4]
	Oleic acid	Leaf	[4]
	*p*-Coumaric acid	Whole plant	[21]
	Ferulic acid	Whole plant	[21]
	Docosapentaenoic acid	Stem	[26]
	Eicosapentaenoic acid		[53]
	Docosahexaenoic acid		[53]
	Catechol		[53]
	Caffeic acid	Aerial part	[54]
	Oxalic acid	Leaf	[2]
	Lonchocarpic acid		[50]

Vitamins	Vitamin A	Leaf	[26]
	Riboflavin	Leaf	[26]
	Niacin	Leaf	[26]
	Pyridoxine	Leaf	[26]
	Vitamin C	Leaf	[26]
	Folates	Leaf	[26]
	Pantothenic acid	Leaf	[26]
	Thiamin	Leaf	[26]
	*α*-Tocopherol	Leaf	[4]
	Hesperidin	Leaf	[55]

Minerals	Phosphorus	Root, stem and leaf	[3]
	Iron	Root, stem and leaf	[3]
	Manganese	Root, stem and leaf	[3]
	Calcium	Root, stem and leaf	[3]
	Copper	Root, stem and leaf	[3]
	Zinc	Leaf	[26]
	Selenium	Leaf	[26]
	Magnesium	Leaf	[26]

Other compounds	Portulacerebroside A (42)	Aerial part	[56]
	*β*-Sitosterol (43)	Aerial part	[1]
	Daucosterol (44)	Aerial part	[1]
	*β*-Carotene	Leaf	[4]
	Glutathione	Leaf	[4]
	Proline	Leaf	[57]
	Melatonin	Leaf	[24]
	1,4-Di-O-acetyl-2,3,5-tri-O-methyl-L-arabinitol	Leaf	[58]
	1,4,5-Tri-O-acetyl-2,3-di-O-methyl-L-arabinitol	Leaf	[58]
	1,5-Di-O-acetyl-2,3,4,6-tetra-O-methyl-D-galactitol	Leaf	[58]
	1,4,5-Tri-O-acetyl-2,3,6-tri-O-methyl-D-galactitol	Leaf	[58]
	1,3,4,5-Tetra-O-acetyl-2,6-di-O-methyl-D-galactitol	Leaf	[58]
	Chlorophyll		[53]
	Tannin		[53]
	Isopimpinellin		[50]
	Robustin		[50]
	Bergapten		[50]
